# Monitoring of asparagine depletion and anti-l-asparaginase antibodies in adult acute lymphoblastic leukemia treated in the pediatric-inspired GRAALL-2005 trial

**DOI:** 10.1038/s41408-018-0084-5

**Published:** 2018-05-22

**Authors:** Jérôme Paillassa, Thibaut Leguay, Xavier Thomas, Françoise Huguet, Marie Audrain, Véronique Lheritier, Christine Vianey-Saban, Cécile Acquaviva-Bourdain, Cécile Pagan, Hervé Dombret, Norbert Ifrah, Nicolas Boissel, Mathilde Hunault-Berger

**Affiliations:** 1 Service des Maladies du Sang, FHU GOAL and CRCINA, INSERM, CHU d’Angers, Angers, France; 2 Service d’hématologie Clinique, CHU de Bordeaux, Bordeaux, France; 3 Service d’hématologie Clinique, CHU de Lyon, Lyon, France; 4 Service d’hématologie Clinique, CHU de Toulouse, Toulouse, France; 5 Laboratoire d’immunologie, CHU de Nantes, Nantes, France; 6 Coordination du Groupe GRAALL, Centre Hospitalier Lyon Sud, Lyon, France; 7 Service Maladies Héréditaires du Métabolisme et Dépistage Néonatal, Centre de Biologie et Pathologie Est, CHU Lyon, Bron, France; 8 Observatoire Régional de la Santé Provence–Alpes–Côte d’Azur (ORS PACA), Marseille, France; 9 Hématologie Adulte, Hôpital Saint Louis, EA-3518, Université Paris 7, Paris, France

In childhood acute lymphoblastic leukemia (ALL), monitoring of asparagine depletion, asparaginase activity or anti-asparaginase antibodies (Abs) is crucial to appreciate the efficacy of l-asparaginase therapy^1,2^. Full asparagine depletion^3^ and high asparaginase activity^4^ are both associated with improved outcomes in both children and adult ALL populations.

In children, the presence of Abs against *Escherichia coli* l-asparaginase has an adverse effect on treatment outcome if a switch toward erwinase (i.e., l-asparaginase from *Erwinia chrysantemi*) is not performed^5^. In adult, the incidence and clinical impact of anti-asparaginase Abs remain to be explored.

In the pediatric-inspired GRAALL trials, adult patients with Philadelphia-negative ALL were exposed to l-asparaginase during induction, consolidation, and delayed intensification. In a pilot study, performed in five French GRAALL centers, asparagine depletion and anti-asparaginase Abs were prospectively investigated in consecutive patients from these trials to determine the incidence of l-asparaginase inactivation.

The GRAALL-2005 and the LL-03 protocols have been previously reported. This phase-III trial aimed to evaluate the impact of high-dose cyclophosphamide during induction and of rituximab in patients with CD20-positive ALL^6^. The LL-03 study evaluated the safety and efficacy of an ALL-type intensive chemotherapy in adult patients with lymphoblastic lymphomas (LL)^7^. The GRAALL-2005 and LL-03 trials shared the same chemotherapy backbone. During induction, *E. coli* l-asparaginase was administered at 6000 IU/m^2^/d intravenous (IV) on D8, D10, D12, then stopped for 8 days to avoid increased toxicity during cyclophosphamide and daunorubicine infusion, and finally resumed on D20, D22, D24, D26, and D28. Patients who failed to reach complete remission (CR) after induction received an idarubicine and high-dose cytarabine-based salvage regimen. Patients in CR received a consolidation course of six 2 weeks blocks including *E. coli* l-asparaginase (10,000 IU/m^2^/infusion) infused on day 3 of blocks 1/4 and on day 16 of blocks 2/5^6^. According to baseline and response criteria, patients in persistent CR received either an allogeneic stem cell transplantation (HSCT) or a late intensification similar to the induction chemotherapy followed by maintenance therapy^8^. In case of allergic reaction, *E. coli* l-asparaginase was switched for erwinase (each dose of *E. coli* l-asparaginase was replaced by one dose of erwinase: 12,000 IU/m^2^ during late intensification, 20,000 IU/m^2^ during consolidation).

Asparagine level and anti-asparaginase Abs were assessed on blood samples at D8, D13, D20, and D29 of induction and late intensification, as well as at the onset of consolidation blocks 1, 2, 4, and 5. Asparagine level was evaluated, after rapid freezing, by reversed-phase liquid chromatographic/tandem mass spectrometric method (full depletion if <2 µmol/L). Anti-asparaginase Abs were detected by ELISA test (threshold of 0.2 optic density (OD) for positivity). Thirty-six patients (median age 35, range 18–55) were included between January 2010 and August 2011. All patients were included in the GRAALL-2005 trial, except for one patient included in the LL-03 trial. All gave informed consent. Their characteristics, outcome, asparagine levels, and anti-asparaginase Abs are described for each of them in Supplementary Figure 1. Within the 5-months follow-up of this study, 1 patient died at D19 of induction because of invasive fungal infection, 35 patients achieved CR and received the planned consolidation course, 5 patients relapsed, 14 underwent HSCT because of high-risk features^6^, and 13 patients received late intensification.

At D8, before the first l-asparaginase infusion, the median asparagine level was 39 µmol/L (range 25–60). At this point, no anti-asparaginase Ab was detected. During induction, a full asparagine depletion was observed at D13, D20, and D26 in 29/30 (97%), 30/30 (100%), and 26/26 (100%) patients who received the planned asparaginase infusions, respectively. The only patient with a detectable asparagine level at D13 (3 µmol/L) was fully depleted at D20, D29, and D36 before consolidation phase. At D29, three patients had detectable asparagine levels but they had only received two or three l-asparaginase infusions because of adverse events. Anti-asparaginase Abs were not detected at D13 and D20 while 1 out of 28 patients (4%) had Abs at D29. This patient did not receive asparaginase infusions after D12 because of severe acute pancreatitis.

Consolidation was initiated as early as possible after CR achievement, depending on recovery from induction toxicity. The interval between D29 of induction and D1 of consolidation ranged from 1 to 30 days (median 10 days). Twenty patients were evaluable for asparagine depletion just before the first consolidation block, among whom 12 (60%) were still fully depleted (Fig. 1a). Interestingly, the 11 patients who begun consolidation between D28 (date of the last asparaginase infusion during induction) and D40 were still fully depleted. Among patients with over 12 days before consolidation initiation, only one was fully depleted. At consolidation onset, 7 out of 20 screened patients (35%) were presented with anti-asparaginase Abs. No correlation was found, however, between the time to consolidation and the presence of Abs. The median time between the first consolidation blocks (Blocks 1 and 2) was 15 days (range 12–51). Six out of the nine patients (67%) evaluated at block 2 onset had full asparagine depletion. All patients (5/5) who received block 2 D15 no later than 15 days after block 1 D2 were still fully depleted in asparagine.

**Fig. 1:**
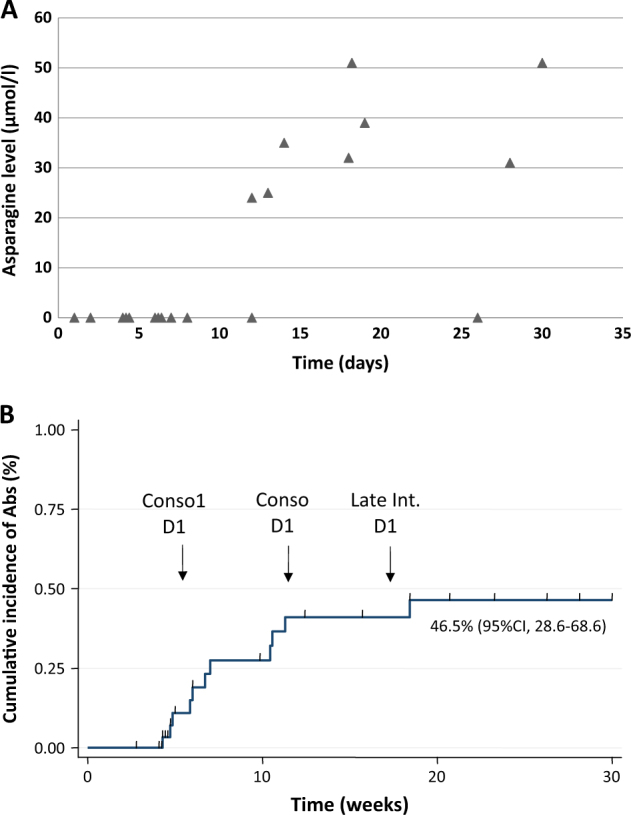
Asparagine depletion duration and cumulative incidence of Abs appearance. **a** Asparagine level at consolidation start according to time between last l-asparaginase infusion (D0) and consolidation. **b** Cumulative incidence of Abs detection. Theoretical time of consolidation 1, consolidation 2, and late intensification are shown

During late intensification, nine patients were evaluable for asparagine depletion. Three patients without previous allergy received *E. coli* l-asparaginase and were fully depleted from D13 to D29 with neither hypersensitivity symptoms nor Abs occurrence. Six patients received erwinase because of previous allergy to *E. coli* l-asparaginase during consolidation (5/6) or encephalitis during induction (1/6). None experienced allergic reaction after erwinase infusions and all were fully depleted in asparagine.

Anaphylactoid reactions occurred in 31% of patients (11/35) during consolidation and late intensification, among them 6 were of grade 3–4 and 9 (82%) with anti-asparaginase Abs. These reactions occurred mostly after the third or fourth infusion of *E. coli* l-asparaginase during consolidation. Types of reactions were variable from rash to anaphylactic shock. Three, two, four, and two patients experienced grade 1, 2, 3, and 4 allergic reaction, respectively.

Anti-asparaginase Abs were detected in 11/26 patients (42%) during consolidation. Among them, 7/11 (64%) had anaphylactoid reactions, 2/11 (18%) had no reaction and were not fully depleted in asparagine (silent inactivation), 2/11 (18%) were not fully evaluated because of HSCT or encephalopathy. The cumulative incidence of Abs positivity, calculated considering relapse, death in first CR, and HSCT as competing events, was 46.5% (95% CI, 28.6–68.6) at 20 weeks (Fig. 1b).Unfortunately, the present pilot study was not powered and designed to address the question of the impact of Abs on relapse incidence. Indeed, only five relapses occurred, one after occurrence of Abs and four in patients without Abs.

We recently reported that patients with CD20-positive ALL who were randomized to receive rituximab had significantly less grade 3–4 hypersensitivity reactions to asparaginase (2/105 in the Rituximab group, 14/104 in the control group)^9^. In the present cohort, 5/31 patients received Rituximab. None of them had clinical signs of hypersensitivity and only 1/5 (20%) was detected with Abs. In contrast, 10/31 patients (32%) who were not exposed to Rituximab presented with hypersensitivity reaction and 13/31 (42%) developed Abs. Due to the small size of our cohort, none of these differences was statistically significant.

After numerous studies in childhood ALL, the present study is the first one to report asparaginase monitoring including anti-asparaginase Abs in adults. Despite the short half-life of l-asparaginase (8–30 h), we observed prolonged depletion in most patients. During induction, all patients who adequately received the l-asparaginase schedule were fully depleted in asparagine, especially at D20, 8 days after the previous asparaginase infusion, and up to 12 days after the last D28 infusion. Likewise, during consolidation phase, l-asparaginase depletion was observed up to 15 days after the previous infusion during consolidation blocks.This may be due in part to the sequential asparaginase infusions while measurement of half-life of l-asparaginase has been performed after one infusion only. However, a false depletion, due to the persistence of circulating asparaginase and post-sampling asparaginase activity cannot be ruled out despite careful freezing of samples. Monitoring asparaginase activity rather than asparagine depletion is currently recommended to appreciate the efficacy of asparaginase and will be evaluated prospectively in the GRAALL-2014 trial.

Due to the postponed sample analysis, physicians were not aware of Abs detection and/or absence of asparagine depletion during treatment. The cumulative incidence of Abs positivity at 20 weeks was 46.5%, in line with previous reports in children/adolescents^10,11^. A strong correlation between the presence of Abs and clinical signs of hypersensitivity was observed and only 2/11 (18%) of patients with anti-asparaginase Abs had silent inactivation. However, such Abs are not always neutralizing and interpretation of their positivity is difficult in the absence of asparaginase activity monitoring. Although the study was not designed to address this question we should keep in mind that none of the patients receiving rituximab experienced hypersensitivity reactions and only one had Abs positivity.

Despite the emergence of new therapies like inotuzumab ozogamicin and blinatumomab in B-cell ALL, asparaginase remain a major drug in the front-line treatment of ALL. The present study highlights the role of therapeutic drug monitoring in the management of adults with ALL, exposed early to l-asparaginase during chemotherapy. Further studies should explore the correlation between asparaginase residual activity and asparagine depletion to improve drug tolerance and efficacy. In the current GRAALL-2014 trial, a prospective monitoring of Abs is performed to switch from *E. coli* l-asparaginase to erwinase in case of clinical and/or silent allergy. The place of rituximab but also of other B-cell targeting therapies like inotuzumab ozogamicin or blinatumomab to decrease the risk of immunization should be further explored.

## Electronic supplementary material

Supplementary Figure

## References

[1] Vrooman LM (2013). Postinduction dexamethasone and individualized dosing of *Escherichia coli* L-asparaginase each improve outcome of children and adolescents with newly diagnosed acute lymphoblastic leukemia: results from a randomized study--Dana-Farber Cancer Institute ALL Consortium Protocol 00-01. J. Clin. Oncol..

[2] Asselin B, Rizzari C (2015). Asparaginase pharmacokinetics and implications of therapeutic drug monitoring. Leuk. Lymphoma.

[3] Jarrar M (2006). Asparagine depletion after pegylated *E. coli* asparaginase treatment and induction outcome in children with acute lymphoblastic leukemia in first bone marrow relapse: a Children’s Oncology Group study (CCG-1941). Pediatr. Blood Cancer.

[4] Wetzler M (2007). Effective asparagine depletion with pegylated asparaginase results in improved outcomes in adult acute lymphoblastic leukemia: Cancer and Leukemia Group B Study 9511. Blood.

[5] Panosyan EH (2004). Asparaginase antibody and asparaginase activity in children with higher-risk acute lymphoblastic leukemia: Children’s Cancer Group Study CCG-1961. J. Pediatr. Hematol. Oncol..

[6] Huguet, F. et al. The upper age limit for a pediatric-like treatment in adult patients with acute lymphoblastic leukemia: report of the Graall-2005 study. *Blood*, in press. 10.1182/blood.2020004919 32321172

[7] Lepretre S (2016). Pediatric-like acute lymphoblastic leukemia therapy in adults with lymphoblastic lymphoma: the GRAALL-LYSA LL03 study. J. Clin. Oncol..

[8] Dhédin N (2015). Role of allogeneic stem cell transplantation in adult patients with Ph-negative acute lymphoblastic leukemia. Blood.

[9] Maury S (2016). Rituximab in B-lineage adult acute lymphoblastic leukemia. N. Engl. J. Med..

[10] Méchinaud F, Corradini NHJ (2006). Asparaginase use: benefits and limits. Corresp. Oncohématol..

[11] van der Sluis IM (2016). *C*onsensus expert recommendations for identification and management of asparaginase hypersensitivity and silent inactivation. Haematologica.

