# Supplementary material for: Monitoring of asparagine depletion and anti-l-asparaginase antibodies in adult acute lymphoblastic leukemia treated in the pediatric-inspired GRAALL-2005 trial
Source: Blood Cancer J. 2018 May 22;8(5):45. doi: 10.1038/s41408-018-0084-5 (PMC5966449; doi:10.1038/s41408-018-0084-5)

Supplemental figure: patient characteristics, outcomes, asparagine depletion and anti-asparaginase Abs evaluation.

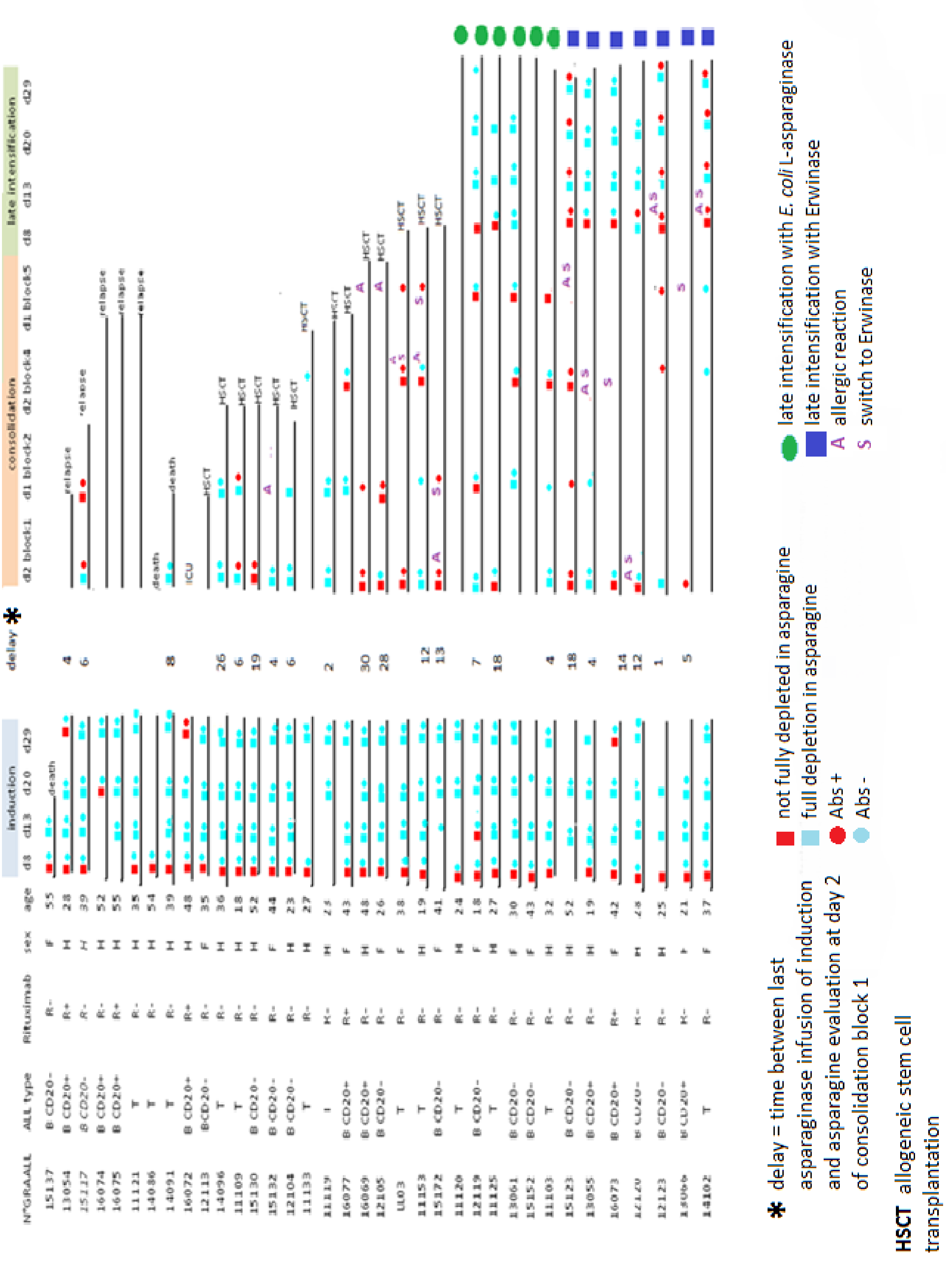

Supplement: Supplementary file 1 — Supplementary Figure [file 41408_2018_84_MOESM1_ESM.pdf]
